# Uric acid and risk of pre-eclampsia: results from a large case–control study and meta-analysis of prospective studies

**DOI:** 10.1038/s41598-023-29651-4

**Published:** 2023-02-21

**Authors:** Claudia C. Colmenares-Mejia, Doris C. Quintero-Lesmes, Paula K. Bautista-Niño, Elizabeth Guío, Maria C. Paez, Mónica Beltrán, David Williams, Kathryn J. Gray, Juan P. Casas, Norma C. Serrano

**Affiliations:** 1 Research Centre, Fundación Cardiovascular de Colombia (FCV), Hospital Internacional de Colombia, Valle de Menzuly Km 7, Piedecuesta, Santander, Bucaramanga, Colombia; 2 Fundación Universitaria Sanitas, Bogotá, Colombia; 3 Biomedical sciences, Universidad del Valle, Cali, Colombia; 4 Faculty of Health Sciences, Universidad Autónoma de Bucaramanga (UNAB), Bucaramanga, Colombia; 5 Department of Public Health, School of Medicine, Universidad Nacional de Colombia, Bogotá, Colombia; 6 Department of Obstetrics and Gynaecology, Universidad Industrial de Santander, Bucaramanga, Colombia; 7 UCL EGA Institute for Women’s Health, University College London Hospital, London, UK; 8 Division of Maternal-Fetal Medicine, Brigham and Women’s Hospital, Boston, MA USA; 9 Center for Genomic Medicine, Massachusetts General Hospital, Boston, MA USA; 10 Massachusetts Veterans Epidemiology Research and Information Center (MAVERIC), VA Boston Healthcare System, Boston, MA USA

**Keywords:** Biomarkers, Health care

## Abstract

To quantify the association between maternal uric acid levels and pre-eclampsia risk in a large collection of primigravid women. A case–control study (1365 cases of pre-eclampsia and 1886 normotensive controls) was conducted. Pre-eclampsia was defined as blood pressure ≥ 140/90 mmHg and proteinuria ≥ 300 mg/24 h. Sub-outcome analysis included early, intermediate, and late pre-eclampsia. Multivariable analysis for pre-eclampsia and its sub-outcomes was conducted using binary and multinomial logistic regression, respectively. Additionally, a systematic review and meta-analysis of cohort studies measuring uric acid levels < 20 weeks of gestation was performed to rule out reverse causation. There was a positive linear association between increasing uric acid levels and presence of pre-eclampsia. Adjusted odds ratio of pre-eclampsia was 1.21 (95%CI 1.11–1.33) for every one standard deviation increase in uric acid levels. No differences in the magnitude of association were observed between early and late pre-eclampsia. Three studies with uric acid measured < 20 weeks’ gestation were identified, with a pooled OR for pre-eclampsia of 1.46 (95%CI 1.22–1.75) for a top vs. bottom quartile comparison. Maternal uric acid levels are associated with risk of pre-eclampsia. Mendelian randomisation studies would be helpful to further explore the causal role of uric acid in pre-eclampsia.

## Introduction

Pre-eclampsia is a multi-system syndrome unique to human pregnancy, which is classically defined by the gestational onset of hypertension and proteinuria^1^. Approximately 4% of first-time pregnancies are affected by preeclampsia, which remains an important worldwide cause of maternal and perinatal mortality and morbidity^2^.

Women with pre-existing hypertension and obesity are predisposed to pre-eclampsia^3,4^, which develops when the placenta fails to adequately invade the maternal uterine decidua^5^. As a consequence, the relatively ischaemic placenta is the source of excess anti-angiogenic compared with pro-angiogenic factors^6,7^. Apart from pre-eclampsia prophylaxis with low-dose aspirin, a better understanding of the aetiology of pre-eclampsia has not been translated into effective therapeutic strategies^8^.

Uric acid is under investigation as a potential causal factor in pre-eclampsia^9,10^. The rationale for investigating uric acid follows from the correlation of elevated uric acid with pre-eclampsia severity (severe hypertension and the HELLP syndrome)^11^. In the general population, an elevated serum uric acid level is associated with two major risk factors for pre-eclampsia, namely hypertension and impaired renal function^12,13^.

During hominoid evolution several mutations occurred in humans and other primates in the gene that encodes for urate oxidase, the enzyme responsible for inactivation of uric acid by oxidation to allantoin. These gene variants raise the level of uric acid in humans compared with other mammals^14^. This has been interpreted as proof of an evolutionary advantage, with high levels of uric acid being linked to several mechanisms affecting antioxidant activity^15^ and blood pressure regulation (through induction of chronic salt sensitivity) observed under low-salt diets, suggested to be present in early hominoids^16^. It has been hypothesized that the increase in salt consumption observed in most modern societies over time transformed this early advantage of chronic salt sensitivity into a deleterious mechanism contributing to the development of hypertension^16^.

In parallel to these phylogenetic and mechanistic studies, several observational studies have described in detail the existence of an association between high levels of uric acid and incidence of hypertension^17,18^. Given the central role of hypertension in pre-eclampsia^19^, some investigators have proposed that high levels of uric acid are involved in the pathophysiological pathway to pre-eclampsia. However, most studies have included too few cases to be conclusive. In addition, the preferred metric of association in previous studies has been the *unadjusted* difference in levels of uric acid between women with pre-eclampsia and healthy pregnant controls^20–23^. This means that confounding by other factors such as maternal age, gestational age, maternal BMI and other unknowns may be an entirely plausible explanation for the observed association.

Making use of one of the largest biological collections of pre-eclampsia, we investigated the association between maternal uric acid levels and the occurrence of pre-eclampsia while taking into account the effect of potential confounders. In addition, and owing to the large sample size (5 times more cases than any previous study), we were able to determine for the first time the nature of the association between uric acid levels and pre-eclampsia risk, and explore whether there was a differential association according to gestational age at presentation of pre-eclampsia. Finally, we investigated whether there was evidence of reverse causation between a diagnosis of pre-eclampsia and elevated uric acid levels by conducting a systematic review and meta-analysis that investigated whether there was an association between uric acid levels in early pregnancy before the clinical onset of pre-eclampsia.

## Results

### Descriptive analysis

A total of 3446 participants (1508 cases and 1938 controls) had available data on uric acid levels. From this, less than 2% of the observations were excluded because of uric acid quantifications reported as unreliable or below detection threshold. Only 3.4% of participants were excluded because of the age limit set to 25 years (Fig. S1).

The final study sample consisted of 1365 cases and 1886 controls (Fig. S1). In both groups, missing data had a low frequency for all variables (< 3%).

Baseline characteristics of GenPE participants are displayed in Table 1. All included participants were primigravida less than 25 years. Overall, most women were of mixed ethnicity and low socioeconomic status, with less than 3% reporting smoking during the current pregnancy, and a very low frequency of multiple pregnancies. The percentage of women in the study that attended at least four antenatal visits (as per WHO recommendations) was 81%. Cases and controls differed regarding the gestational age at delivery, with pre-eclampsia cases delivering on average three weeks earlier than controls; for cases there was a greater proportion who had a mother or sister affected by pre-eclampsia (Table 1). Cases were older than controls. New-born weight was lower in cases and in the group classified in the highest maternal uric acid quintile (Fig. S2 and Table S1).

**Table 1:** Clinical and demographic characteristics of GenPE participants.

Variable	Controls (n = 1886)	Pre-eclampsia (n = 1365)	Difference (95%CI)^a^
Age (years), mean (SD)	18.7 (2.8)	19.3 (3.1)	− 0.6 (− 0.80, − 0.39)
Gestational age at recruitment (weeks), mean (SD)	39.1 (1.1)	36.6 (3.4)	2.5 (2.38, 2.76)
Systolic Blood Pressure (mmHg), mean (SD)	110.9 (7.6)	147.7 (11.6)	− 36.8 (− 37.5, − 36.0)
Diastolic Blood Pressure (mmHg), mean (SD)	68.5 (6.3)	96.4 (8.8)	− 27.8 (− 28.4, − 27.3)
Antenatal visits, median (IQR)	6.0 (4.0, 7.0)	6.0 (4.0, 7.0)	0.0 (− 0.16, 0.16)

			OR (95%CI)^a^
Ethnicity, N (%)			
Mixed	1410 (74.7%)	969 (70.9%)	Reference
White Hispanic	294 (15.5%)	237 (17.3%)	1.17 (0.97, 1.41)
Afro-Caribbean	153 (8.1%)	132 (9.6%)	1.25 (0.98, 1.60)
Amerindians	6 (0.3%)	13 (0.9%)	3.15 (1.19, 8.33)
Missing	23 (1.2%)	13 (0.9%)	
Socioeconomic status, N (%)			
Medium–High	78 (4.1%)	60 (4.4%)	Reference
Low	1768 (93.7%)	1280 (93.7%)	0.94 (0.66, 1.32)
Missing	40 (2.1%)	25 (1.8%)	
Mother with PE, N (%)			
No	1363 (72.2%)	874 (64.0%)	Reference
Yes	130 (6.8%)	209 (15.3%)	1.11(1.02, 1.21)
Missing	6 (0.3%)	12 (0.8%)	
Sister with PE, N (%)			
No	871 (46.0%)	663 (49.1%)	Reference
Yes	94 (4.9%)	130 (9.5%)	1.14 (0.98, 1.31)
No sister with pregnancy	809 (42.9%)	483 (35.3%)	
Missing	16 (0.8%)	15 (1.1%)	
Infections, N (%)			
No	665 (35.2%)	469 (34.3%)	Reference
Yes	1189 (63.0%)	868 (63.5%)	1.04 (0.90, 1.19)
Missing	4 (0.2%)	5 (0.3%)	
Smoking, N (%)			
No	1828 (96.9)	1328 (97.2%)	Reference
Yes	49 (2.6%)	24 (1.7%)	0.67 (0.41, 1.10)
Missing	9 (0.4%)	13 (0.9%)	
Multiple pregnancy, N (%)			
No	1882 (99.7%)	1330 (97.4%)	Reference
Yes	4 (0.2%)	35 (2.5%)	12.38 (4.39, 34.91)
Missing	0	0	

PE, pre-eclampsia; IQR, interquartile range.

^a^Mean differences calculated with t-test with unequal variances for continuous variables. Difference in medians calculated with median regression. For categorical variables, unadjusted ORs were calculated through logistic regression with controls as reference group. Infections: vaginosis, urinary tract infections, sexual transmitted diseases and others.

### Association analysis

**Figure 1:**
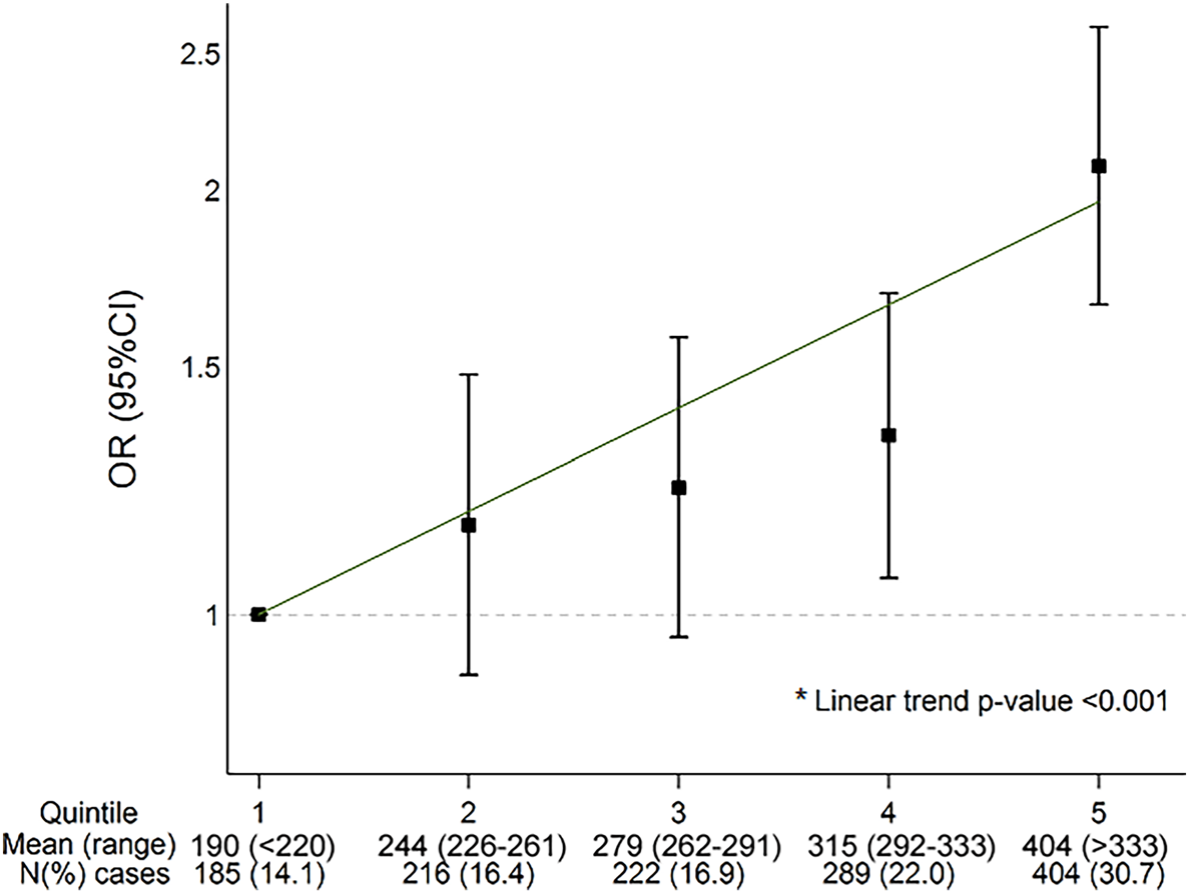
Association between uric acid quintiles and presence of pre-eclampsia after adjustment for maternal age. Mean (range) for quintiles given in μmol/L.

A positive linear relationship, with no evidence of a threshold, was observed between quintiles of uric acid and the presence of the pre-eclampsia; the p-value for a linear trend across quintiles was < 0.001 (Fig. 1).

The logistic model evaluating the association of uric acid as a continuous variable (one SD increase unit) and pre-eclampsia estimated an unadjusted OR of 1.31 (95%CI 1.22, 1.41), which was gradually attenuated by the sequential inclusion of potential confounders until obtaining a fully-adjusted OR of 1.21 (95%CI 1.11, 1.33) for one SD increase in uric acid levels. From the list of potential confounders included in the final model, gestational age was the most influential on the estimates (Likelihood ratio for gestational age = 784.56 in finally adjusted model) (Table 2).

**Table 2:** Uric acid levels and presence of pre-eclampsia with progressive adjustment of logistic regression models.

Progressive adjustment	Cases	Controls	OR (95% CI)^a^	p-value
Unadjusted	1,365	1,886	1.31 (1.22, 1.41)	< 0.001
Plus maternal age	1,365	1,886	1.30 (1.21, 1.40)	< 0.001
Plus gestational age	1,352	1,886	1.19 (1.10, 1.30)	< 0.001
Plus race	1,340	1,863	1.20 (1.10, 1.30)	< 0.001
Plus recruitment place	1,340	1,863	1.21 (1.11, 1.32)	< 0.001
Plus recruitment date	1,340	1,863	1.23 (1.12, 1.34)	< 0.001
Plus smoking	1,328	1,854	1.23 (1.13, 1.35)	< 0.001
Plus multiple pregnancy	1,328	1,854	1.23 (1.13, 1.35)	< 0.001
Plus low SES	1,304	1,815	1.21 (1.11, 1.33)	< 0.001
Plus infections	1,301	1,812	1.21 (1.11, 1.33)	< 0.001

SES, Socioeconomic status.

^a^OR indicates the change in odds of being a case for each 1 SD increase in uric acid levels.

**Figure 2:**
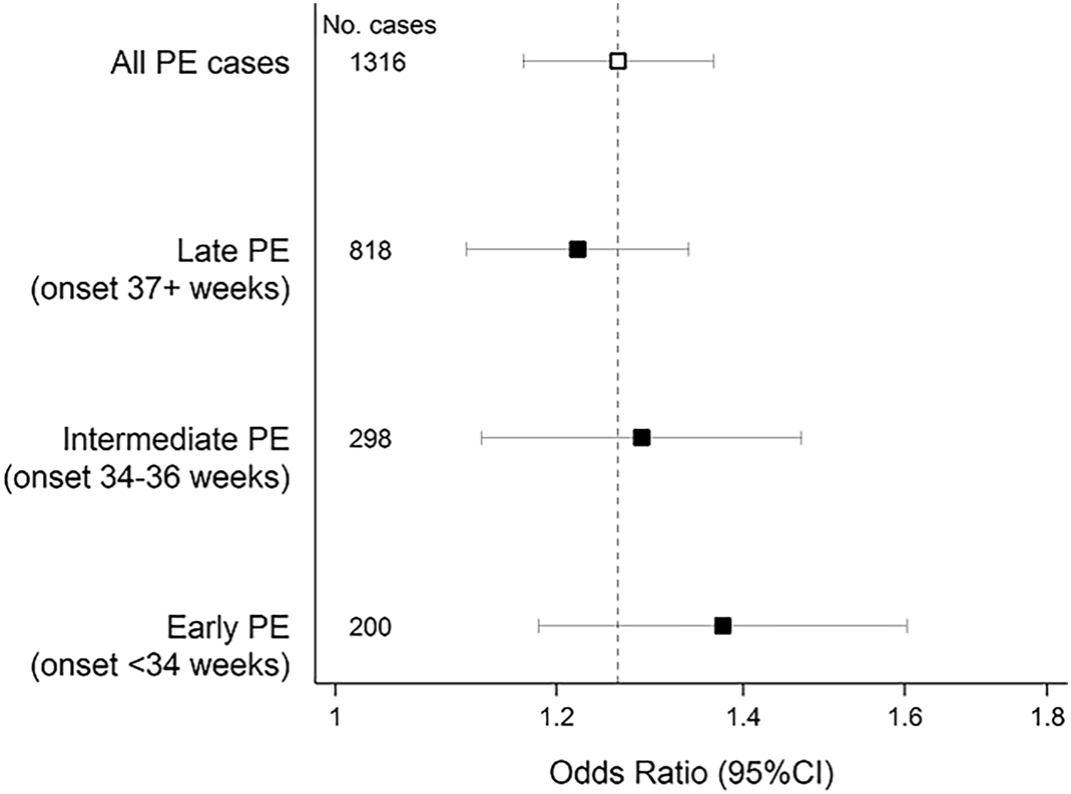
Serum uric acid levels and presence of early, intermediate and late pre-eclampsia in adjusted models* (*Multinomial regression model for early, intermediate and late pre-eclampsia adjusted for maternal age, recruitment centre, ethnicity, recruitment date, multiple pregnancy, smoking, socioeconomic position and infections during pregnancy. Gestational age was not included in the model because this variable defines the sub-outcomes. Results from the logistic model independent of the gestational age of onset is presented for comparison (hollow square). Odds ratios are reported on a logarithmic scale.).

Subset analysis according to gestational age of disease onset suggested a tendency for a stronger association of uric acid levels among women with onset of pre-eclampsia before 34 weeks [adjusted OR 1.38, (95%CI 1.18, 1.60)] compared to those with pre-eclampsia onset between 34 and 37 weeks [adjusted OR 1.29 (95%CI 1.13, 1.47)], and those with pre-eclampsia onset after 37 weeks [adjusted OR 1.22 (95%CI 1.11, 1.34)] (Fig. 2). However, the confidence intervals overlapped considerably across all 3 gestational-age groups.

The test for effect modification according to duration of sample storage showed no evidence of a differential effect of uric acid in models, with p > 0.200 in both minimal (including maternal age, gestational age, recruitment centre, and ethnicity) and fully-adjusted (previous model plus recruitment date, multiple pregnancy, smoking, socioeconomic position and infections during pregnancy) models.

### Systematic review and meta-analysis

**Figure 3:**
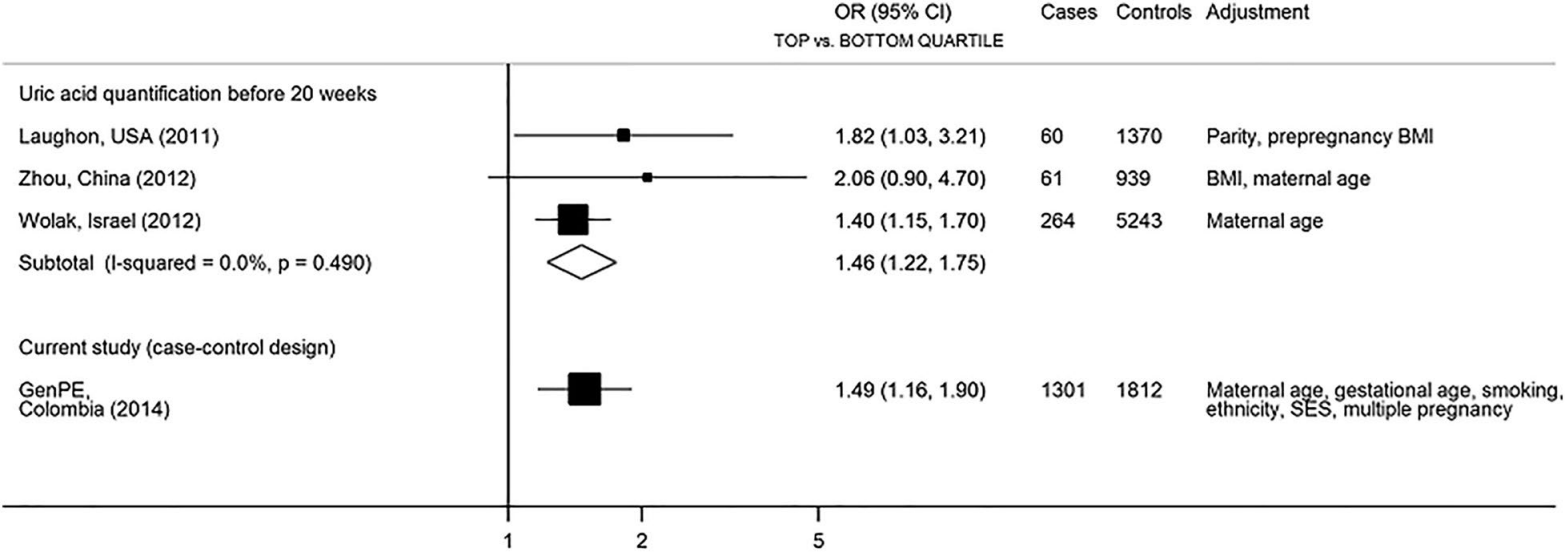
Summary estimates for a comparison between top and bottom quartiles of uric acid for prospective studies with uric acid measured in early pregnancy compared to GenPE results. The p value corresponds to heterogeneity hypothesis testing among studies included in the meta-analysis.

From 396 publications originally identified, 9 studies were reviewed in full text, and three observational studies were included in meta-analysis as they reported the risk of pre-eclampsia as an outcome and presented estimates (i.e. odds ratios for a top *vs.* bottom quartile comparison) that could be meta-analysed^20,24,25^ (Fig. S3, Table S2). The characteristics of the three studies are presented in Table S3 and quality assessment in Table S4. The meta-analysis included 385 cases and 7006 non-cases and showed strong evidence for women in the highest quartile of uric acid distribution measured in early pregnancy having a greater risk of developing pre-eclampsia, with a summary OR for a top vs. bottom quartile comparison of 1.46 (95%CI 1.22–1.75). A comparison of the top vs. bottom quartile of uric acid levels in our case–control study gave an OR of pre-eclampsia of 1.49 (1.16, 1.90), which is very similar to the results observed for uric acid levels measured in early pregnancy before the development of clinical pre-eclampsia (Fig. 3).

## Discussion

### Main findings

This study assessed the association between maternal uric acid levels with a diagnosis of pre-eclampsia in one of the largest biological collections of pre-eclampsia in the world. In this analysis, elevated uric acid levels were associated with increased odds of having pre-eclampsia; this association had a linear relationship without an observable threshold that was robust to adjustment for a comprehensive set of possible confounders. Similar results have been found by Hassen et al.^24^ who reported that being preeclamptic increased serum uric acid levels by a factor of 2.37, and by Shakarami et al.^25^ that after controlling for maternal age, gestational age and BMI, chances of developing pre-eclampsia increased by 1.98.

Although a sensitivity analysis suggested a stronger association for pre-eclampsia-onset before 34 + 0 weeks compared with 34 to 36 + 6 weeks and after 37 + 0 weeks, the confidence intervals for these three gestational age groups overlapped. A similar result was reported by Watanabe et al.^26^ who found that uric acid levels in women with both early and late pre-eclampsia were significantly different from the control group, but levels among women with late pre-eclampsia, although slightly higher, were not statistically different from early pre-eclampsia. Uric acid levels and time of onset is also been reported by^27^ Given the large sample analysed in GenPE, the lack of effect of uric acid level on gestational age of onset of preeclampsia, is likely to be real, rather than a lack of power to detect it.

A systematic review and meta-analysis of three studies measuring uric acid before the clinical onset of pre-eclampsia found a similar odds ratio of pre-eclampsia in later pregnancy to that observed in our case–control study. Thus, reverse causation is not likely to entirely explain the reported association.

### Strengths and limitations

This study has several stregnths; in first place, sample size allowed subgroup analysis including uric acid quintiles and time of onset outcome; which is of great importance for clinical decisions. In addition, the focus of this paper seeks to complement evidence on potential causal effect of uric acid, and not only to report an association analysis between an exposure and outcome, as most observational studies do; from epidemiological perspective, we address the criteria of dose response and strength of association through design and statistical analysis, temporality and consistency through systematic review and meta-analysis. In this way, given that association between uric acid levels and pre-eclampsia may be affected by confounding and/or reverse causation, which are known disadvantages of observational studies, we took several steps to decrease the probability of the observed association being explained by confounding. We adjusted for important potential confounders and found that the association between uric acid and pre-eclampsia persisted independently of these factors. However, residual confounding cannot be ruled out because some information that might affect uric acid levels such as daily fructose, protein intake and pre-pregnancy weight were not recorded due to study design.

On the other hand, GenPE recruited term pregnancies as controls in order to avoid a misclassification bias; preterm controls could became cases at a later pregnancy stage and affect the strength of association or effect direction. However, despite adjustment for gestational age, residual confounding remains because uric acid levels could be lower at earlier gestational age due to estrogen effect, expanded blood volume and increased glomerular filtration rate^9^. Nonetheless, as uric acid levels rise in the third trimester of healthy pregnancy, the differences between preeclamptic pregnancies and controls found in this study are strengthened given that cases were delivered at an earlier gestational age.

### Interpretation

**Figure 4:**
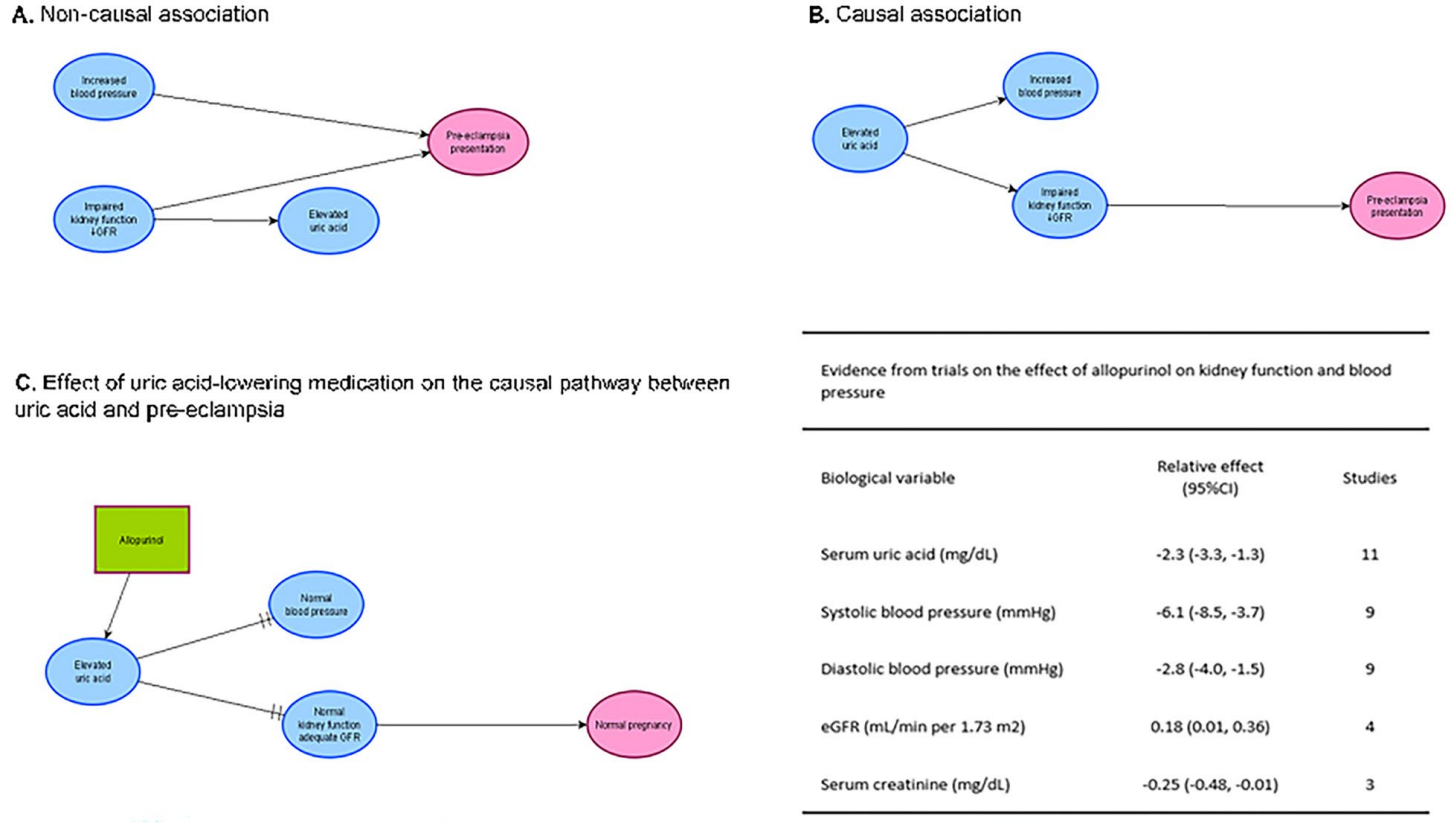
Proposed scenarios for the role of uric acid in pre-eclampsia aetiology. In (**A**), increased uric acid is associated with pre-eclampsia but does not contribute to the pathogenesis of the disease, while in (**B**) uric acid acts as a causal factor for pre-eclampsia. In (**C**), the effect of the uric acid-lowering drug allopurinol^36,37,45,46^^35,36,44,45^ interrupts the causal pathway (as evidenced in non-pregnant individuals).

The association between maternal uric acid and pre-eclampsia has been studied for almost a century^28^, but there are still no firm conclusions about its role^11,26,29^. The elevation of uric acid observed in pre-eclampsia has commonly been attributed to decreased uric acid clearance that occurs as a consequence of the reduced glomerular filtration rate due to pre-eclampsia itself. In this scenario, hyperuricemia is considered a marker of the disease as opposed to a causal factor (Fig. 4A). As a disease marker, uric acid has been studied as a predictor of pre-eclampsia and disease severity, with contradictory findings^11,30^. NICE guidelines from 2019 (https://www.nice.org.uk/guidance/ng133) concluded that evidence was insufficient to advise routine uric acid screening in pregnancy for pre-eclampsia prediction. The magnitude of the association between pre-eclampsia and increased uric acid estimated in our study suggests that uric acid, in isolation, is not useful to identify women at high risk of pre-eclampsia.

Nevertheless, the recent re-evaluation of the role of uric acid in the pathogenesis of hypertension and endothelial and renal dysfunction, which are all characteristic features of pre-eclampsia, has renewed the interest in the role of uric acid in pre-eclampsia aetiology. In this scenario, hyperuricemia is considered a causal contributor to pre-eclampsia (Fig. 4B), but the mechanisms for this effect are still unknown.

In vitro studies provide some support for the hypothesis that uric acid may be involved in pre-eclampsia aetiology, but these studies are extremely limited given the lack of reproducibility of results in epidemiological studies^31–34^. While observational data is also limited in order to establish causal associations, evidence from randomised clinical trials (RCT) is close to non-existent for this association, as the only published RCT for a uric acid lowering intervention (e.g. allopurinol) during pregnancy was a small study not designed to address the effect of the intervention on pre-eclampsia^35^.

There are nevertheless RCTs in the general population evaluating the effect of allopurinol on outcomes that are diagnostic characteristics of pre-eclampsia: impaired kidney function (e.g. decreased glomerular filtration rate) and increased blood pressure. These trials have shown a reduction in uric acid levels with the use of allopurinol that correlates with increased eGFR and decreased blood pressure^36,37^. If the effects of allopurinol are mediated only through reduction of uric acid levels, this may suggest relevant effects of uric acid and a role for reducing serum uric acid levels for the prevention of pre-eclampsia (Fig. 4C). Given that this evidence comes from RCTs conducted mostly in middle-aged men and women, some of them with established renal and cardiovascular morbidity, the results might not be relevant to pregnant women at risk of pre-eclampsia. Nevertheless, there is evidence to favour evaluating a causal effect of uric acid in pre-eclampsia. Knowledge of the genetics of uric acid metabolism^38,39^ would support a Mendelian randomisation study in pregnant women. This has been done for the association between uric acid and other outcomes^40–42^ and could help determine whether uric acid is a causative factor in pre-eclampsia. For this approach, a large sample size is essential, and consortia such as the InterPregGen collaboration^43^ offer a rich source of data for this type of analysis. If supporting evidence is generated, an RCT of uric acid-lowering therapy such as allopurinol may provide evidence for prophylactic efficacy in pre-eclampsia prevention.

## Conclusion

Observational data is suggestive of a role for uric acid in pre-eclampsia development. In order to confirm or refute the involvement of maternal uric acid levels in the aetiology of pre-eclampsia, evidence from randomised clinical trials evaluating uric acid-lowering interventions is required. Future Mendelian randomisation analyses, or other genetic or multiomics approaches, could help inform the planning of such trials.

## Material and methods

### Study design and participants

GenPE (Genetics and Preeclampsia) is a multicentre case–control study conducted in eight Colombian cities. Young (< 25 years) primigravid (pregnant for the first time) women were recruited at the time of delivery between December 2000 and February 2012. Age limit was set in order to decrease the probability of recruiting women with previously undiagnosed chronic conditions.

A case of pre-eclampsia was defined as new-onset hypertension (blood pressure ≥ 140/90 mmHg) and proteinuria (≥ 300 mg in 24 h or ≥ 1 + dipstick reading in a random urine sample with no evidence of urinary tract infection) after the 20th week of gestation. Controls were healthy normotensive pregnant women without proteinuria recruited at term gestation (≥ 37 weeks). Women with a history of arterial hypertension, diabetes mellitus (or gestational diabetes), and renal or autoimmune diseases were excluded from the study. Cases and controls were not matched for any variable.

An outcome committee met periodically to validate the inclusion of participants by reviewing medical records. Data used in this study come from confirmed cases and controls only.

Pre-eclampsia sub-phenotypes were defined according to time of onset: early pre-eclampsia (presentation before 34 + 0 weeks), intermediate pre-eclampsia (presentation between 34 weeks and 36 + 6 weeks), and late pre-eclampsia for presentation after 37 + 0 weeks of gestation.

### Data collection

A verbal interview with a structured questionnaire was conducted by trained personnel for every woman agreeing to participate in order to ascertain demographic and clinical data such as maternal age, ethnicity, socioeconomic status ranging from 0 to 6 (the most deprived scoring 0, the most affluent scoring 6 and low socioeconomic status defined as below 3)^44^, smoking, infections during the index pregnancy (e.g. vaginosis, urinary tract infections, sexual transmitted diseases and others), family history of pre-eclampsia in mother and sisters, and gestational age at recruitment. Data recorded from clinical records included two values of blood pressure (taken minimum 4 h apart), proteinuria level, and medical data on the offspring. Blood pressure was measured in the right arm in a seated position after five minutes rest with mercury sphygmomanometers. Given the difficulty in linking antenatal care information, no retrospective clinical record data was obtained.

At recruitment, all participants signed written informed consent including the use of their information for derived projects from GenPE study. The project was approved by the Research Ethics Committee of Universidad Autónoma de Bucaramanga—UNAB.

### Blood sampling and biochemical analysis

Blood samples were drawn at recruitment (at delivery) from the antecubital vein using tubes without anticoagulant (Becton Dickinson, USA). Samples were allowed to clot and subsequently centrifuged for 10 min. The obtained serum was transferred to vials in fractions of 300 μL and stored at − 80 °C until the uric acid assay was performed. Uric acid quantification took place between June 2010 and July 2011 using an enzymatic colorimetric method with sensitivity of 20.8 μmol/L and detection range of 129.1–1373.1 μmol/L in the RxImola automated system (Randox, Antrim, UK). Laboratory technicians were blinded to participants’ disease status. Replication of uric acid measurement was performed in 200 random samples for quality control.

### Statistical analyses

#### Descriptive analysis

Continuous variables were described as mean and standard deviation (SD) with previous verification of a normal distribution, and categorical variables were described as counts and proportions. The proportion of missing values for each variable included in analysis was reported. Unadjusted differences between cases and controls were quantified using the *t* test for two samples with unequal variance for continuous variables and unadjusted odds ratios (OR) with 95% confidence intervals (95%CI) for categorical variables.

A total of 61 samples reporting unreliable uric acid measurements or values below/above detection limits were excluded from the analysis. In addition, measurements of uric acid above or below four SDs (seven cases and seven controls) were considered outliers and were consequently excluded from the association analysis; this was deemed appropriate given that their inclusion could lead to an erroneous interpretation of the nature of the association of uric acid levels with pre-eclampsia risk.

#### Association analysis

In order to describe the observed dose–response relationship between uric acid and pre-eclampsia risk, uric acid levels were categorized into quintiles according to the distribution in the control group (≤ 220 μmol/L, 226–261 μmol/L, 262–291 μmol/L, 292–333 μmol/L, and ≥ 333 μmol/L), and a test for linear trend across quintiles was performed after adjusting for maternal age in a logistic regression model. For association analysis, uric acid levels, which followed an approximate normal distribution, were standardized before evaluating the metabolite as a continuous variable. Multinomial regression was preferred for the analysis of pre-eclampsia sub-phenotypes.

Logistic and multinomial regressions models were adjusted progressively by maternal age, gestational age, recruitment centre, ethnicity, recruitment date, multiple pregnancy, smoking, socioeconomic position and infections during pregnancy as possible confounders. In multinomial regression analysis, gestational age was not included as this variable defined the sub-outcomes.

Subgroup analysis was carried out according to the time of storage of the samples, from the sampling date to the measurement of uric acid (categorized as ≤ 5 or > 5 years) to assess for possible effect modification in the association between uric acid levels and pre-eclampsia risk. Effect modification was tested with a likelihood ratio test comparing regression models with and without an interaction term (in a multiplicative scale) for uric acid levels and storage time.

#### Systematic review and meta-analysis

In order to present results of GenPE study in the context of current evidence, a systematic literature search (Pubmed, Scopus and Google scholar) for studies reporting the association between uric acid levels in serum or plasma and pre-eclampsia was performed, restricting the inclusion criteria for meta-analysis to those studies that obtained the blood samples before 20 weeks of gestation (i.e. before the clinical onset of pre-eclampsia) and reported relative measures of association. PRISMA guidelines were followed for systematic literature search and reporting results (Supplementary data). Estimates from adjusted models were preferred and were meta-analysed in a top vs. bottom quartile scale using random-effects models.

#### Effect of uric acid-lowering therapies on blood pressure renal outcomes

Summary estimates (weighted mean differences; 95%CI) for the effect of uric acid-lowering therapies, mainly allopurinol, on systolic and diastolic blood pressure and renal outcomes (serum creatinine and glomerular filtration rate) in randomised clinical trials were obtained after extracting and combining information of individual studies included in previous systematic reviews and meta-analyses (Supplementary data).

Data analysis was performed with Stata 13 (StataCorp, USA).

This study was carried out according to principles in Declaration of Helsinki; in this way ethics aproval was granted by Research Ethics Committee at Universidad Autónoma de Bucaramanga (December 10th 2008, #0048/2008).

### Ethics approval

Ethics aproval was granted by Research Ethics Committee at Universidad Autónoma de Bucaramanga (December 10th 2008, #0048/2008).

## Supplementary Information

Supplementary Information.

## Data Availability

The datasets used and/or analysed during the current study available from the corresponding author on reasonable request.
