# Supplementary material for: Uric acid and risk of pre-eclampsia: results from a large case–control study and meta-analysis of prospective studies
Source: Sci Rep. 2023 Feb 21;13:3018. doi: 10.1038/s41598-023-29651-4 (PMC9944921; doi:10.1038/s41598-023-29651-4)
Supplement: Supplementary file 1 — Supplementary Information. [file 41598_2023_29651_MOESM1_ESM.docx]

**Uric Acid and Risk of Pre-Eclampsia: Results from a Large Case-Control Study and Meta-Analysis of Prospective Studies**

**Methods**

**Meta-analysis of cohort studies**

We performed a systematic search in Pubmed, Scopus and Google scholar using uric acid AND preeclampsia as search terms, without date or language restriction to identify studies with uric acid measurements in the first 20 weeks of pregnancy. The search was done during November of 2013 and updated in December of 2021. No additional studies were identified in second search.

**Selection criteria**

Observational cohort studies were included if they assessed the association between serum or plasma uric acid levels measured before 20 weeks of gestation and risk of pre-eclampsia at the end of pregnancy. Studies were excluded if they not reported Odds Ratios as measure of association.

**Data extraction**

One of the authors performed data extraction on a excel template designed according to variables of interest and data was reviewed by a second author. Disagreements were resolved between the two authors.

**Quality assessment**

Methodological and quality assessment of cohort studies was carried out with Newcastle-Ottawa Scale (NOS) (1)

**Statistical analysis**

For meta-analysis, pooled Odds Ratios for pre-eclampsia and 95% CI were estimated using random effects for a comparison between top and bottom quartiles of uric acid measured in the first 20 weeks of pregnancy.

**References**

1. Wells G, Shea B, O’Connell D, Peterson J, Welch V, Losos M. The Newcastle–Ottawa Scale (NOS) for assessing the quality of nonrandomised studies in meta-analyses. [Internet]. 2014 [cited 2022 Jun 19]. Available from: https://www.ohri.ca//programs/clinical_epidemiology/oxford.Asp

**Effect of uric acid-lowering therapies on blood pressure renal outcomes**

A search was performed in PubMed without restrictions to identify previous systematic reviews or meta-analysis of randomised clinical trials reporting the effect of uric acid-lowering therapies (mainly Allopurinol) on blood pressure and renal outcomes.

Information from individual studies included in the four reviews identified (1–4) was extracted and combined, when appropriate, as weighted mean differences between the active and treated groups at the end of the trials.

Summary estimates were presented for the effect of Allopurinol on uric acid levels (mg/dL), systolic and diastolic blood pressure (mmHg), estimated glomerular filtration rate (eGFR; mL/min per 1.73 m2), and serum creatinine (mg/dL).

**References**

1. Agarwal V, Hans N, Messerli FH. Effect of allopurinol on blood pressure: a systematic review and meta‐analysis. The Journal of Clinical Hypertension. 2013;15(6):435–42.

2. Bose B, Badve SV, Hiremath SS, Boudville N, Brown FG, Cass A, et al. Effects of uric acid-lowering therapy on renal outcomes: a systematic review and meta-analysis. Nephrology Dialysis Transplantation. 2013;29(2):406–13.

3. Kabul S, Shepler B. A review investigating the effect of allopurinol on the progression of kidney disease in hyperuricemic patients with chronic kidney disease. Clin Ther. 2012;34(12):2293–6.

4. Kanbay M, Siriopol D, Nistor I, Elcioglu OC, Telci O, Takir M, et al. Effects of allopurinol on endothelial dysfunction: a meta-analysis. Am J Nephrol. 2014;39(4):348–56.

**Figure S1. Flowchart of participants recruited in GenPE study between December 2000 and May 2012.**

Total number of women recruited in the GenPE study by May 2012 (N=9032)

-4011 cases

-5021 controls

Records excluded after review by outcome committee (N=702)

-309 failed criteria for cases

-316 failed criteria for controls

-77 not classifiable

From validated sample (N=8330), number of blood samples selected for uric acid quantification

-1508 cases

-1937 controls

Samples excluded from analysis (N=75)

-Quantification reported as not reliable n= 50

-Below detection limit n = 11

-Values above 4SD n=14

Records with uric acid quantification available for analysis (N=3265)

-1372 cases

-1891 controls

Records excluded for maternal age >25 years old (n = 120)

Final sample

1365 cases

1886 controls

**Figure S2. New-born weight and uric acid levels according to maternal status**

**Table S1. Mean new-born weight according to uric acid quintiles by maternal status**

| **Uric acid quintile in mother** | **n** | **New-born weight in controls (gr)** | **n** | **New-born weight in cases (gr)** |
| --- | --- | --- | --- | --- |
| 1 (≤220 umol/L) | 397 | 3160 (445) | 206 | 2771 (686) |
| 2 (226-261 umol/L) | 363 | 3164 (423) | 211 | 2670 (754) |
| 3 (262-291 umol/L) | 357 | 3164 (420) | 215 | 2609 (759) |
| 4 (292-333 umol/L) | 364 | 3147 (397) | 242 | 2578 (741) |
| 5 (≥333 umol/L) | 361 | 3148 (422) | 383 | 2555 (754) |

Figure S3. Flow chart for selection of studies included in meta-analysis.

Other Outcomes (fetal or perinatal, gestational hypertension, composite maternal outcomes, pre-eclampsia severity uric acid by BMI): 3

No association estimates (mean levels comparison, AUC/ROC): 7

Total citations retrieved by electronic searches (n = 1.189)

Studies screened in full text (n = 13)

Studies in meta-analysis (n = 3)

Citations reviewed by title and/or abstract (n = 770)

Studies not fulfilling inclusion criteria

(For design: Systematics reviews, Case-control, diagnostic performance, case reports, reviews, cohorts for second and third trimester, animal studies, editorials, n = 143)

(Other markers different from uric acid, n = 614)

Duplications removed

(n = 419)

**Table S2. Reasons for excluded studies**

| Author, year | Title | Cohort | Uric acid <20 week | Outcome | Reporting OR |
| --- | --- | --- | --- | --- | --- |
| de Jogn, 1997 | Decreased first trimester uric acid production in future preeclamptic patients | Prospective cohort | Not clear | Severity of pregnancy induced hypertension | - |
| Merviel, 1998 | Lone hyperuricemia during pregnancy: Maternal and fetal outcomes | Prospective cohort | Yes | Pre-eclampsia | No |
| Wakwe, 1999 | Estimation of plasma uric acid in pregnancy induced hypertension (PIH). Is the test still relevant? | Prospective cohort | Yes | Pre-eclampsia | No |
| Salako, 2004 | Serum albumin, creatinine, uric acid and hypertensive disorders of pregnancy | Prospective cohort | Yes | Pre-eclampsia | No |
| Hawkins, 2012 | ﻿Plasma uric acid remains a marker of poor outcome in hypertensive pregnancy: A retrospective cohort study | Retrospective cohort | Not clear | Composite outcome | - |
| Tangeras, 2015 | Distinct First Trimester Cytokine Profiles for Gestational Hypertension and Preeclampsia | Prospective cohort | Yes | Pre-eclampsia and gestational hypertension | No |
| Osakwe, 2015 | ﻿The predictive value of serum uric acid for the occurrence, severity and outcomes of pre-eclampsia among parturients at nnewi, nigeria. | Prospective cohort | Yes | Pre-eclampsia | No |
| Cheng, 2015 | Serum uric acid may not be involved in the development of preeclampsia | Prospective cohort | Yes | Pre-eclampsia | Yes. OR corresponded to the odds of being a case for increase in one unit of uric acid (Not quintiles comparison) |
| Rezk, 2018 | First versus second trimester mean platelet volume and uric acid for prediction of preeclampsia in women at moderate and low risk | Prospective cohort | Yes | Pre-eclampsia | OR for accuracy performance of uric acid as diagnostic test |
| Čabarkapa, 2018 | Serum magnesium level in the first trimester of pregnancy as a predictor of pre-eclampsia – a pilot study | Prospective cohort | Yes | Pre-eclampsia | No |

Table S3. Characteristics of studies with uric acid measured before 20 weeks evaluating the risk of pre-eclampsia (PE).

| **Author (year)** | **Geographical location (ethnicity)** | **Cases/**  **non-cases** | **Case definition**  **BP: blood pressure**  **PU: proteinuria** | **Maternal age (years): cases / controls** | **Study design** | **Uric Acid assay;**  **units** |
| --- | --- | --- | --- | --- | --- | --- |
| Zhou (2012) | China  (Asian) | 61/939 | BP > 140/90 &  PU >300mg | 29.6/28.6 | Prospective | Colorimetric assay; umol/L |
|  |  |  |  |  |  |  |
| Wolak (2012) | Israel  (Middle East) | 264/5243 | No specific values; referred to as hypertension + proteinuria | Not reported | Retrospective cohort | Not reported; mEq/L |
|  |  |  |  |  |  |  |
| Laughon (2011) | United States  (Caucasian,  African-American) | 60/1370 | BP > 140/90 &  PU >300mg | Not reported | Prospective | Colorimetric assay; mg/dl |

Table S4. Quality assessment of included studies

| **Criteria** | **Zhou (2012)** | **Wolak (2012)** | **Laughon (2011)** |
| --- | --- | --- | --- |
| **Selection** |  |  |  |
| 1) Representativeness of the exposed cohort |  |  |  |
| A | * | * | * |
| B |  |  |  |
| C |  |  |  |
| D |  |  |  |
| 2) Selection of the non exposed cohort |  |  |  |
| A | * | * | * |
| B |  |  |  |
| C |  |  |  |
| 3) Ascertainment of exposure |  |  |  |
| A | * | * | * |
| B |  |  |  |
| C |  |  |  |
| D |  |  |  |
| 4) Demonstration that outcome of interest was not present at start of study |  |  |  |
| A | * | * | * |
| B |  |  |  |
| **Comparability** |  |  |  |
| 1) Comparability of cohorts on the basis of the design or analysis |  |  |  |
| 1. Low levels of uric acid (top bottom quartile) | * | * | * |
| B |  |  |  |
| **Outcome** |  |  |  |
| 1) Assessment of outcome |  |  |  |
| A |  |  |  |
| B | * | * | * |
| C |  |  |  |
| D |  |  |  |
| 2) Was follow-up long enough for outcomes to occur |  |  |  |
| A | * | * | * |
| B |  |  |  |
| 3) Adequacy of follow up of cohorts |  |  |  |
| A |  |  | * |
| B | * |  |  |
| C |  |  |  |
| D |  | * |  |


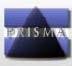
**PRISMA 2020 Checklist**

| **Section and Topic** | **Item #** | | **Checklist item** | **Location where item**  **is reported** | | |
| --- | --- | --- | --- | --- | --- | --- |
| **TITLE** | | | |  | | |
| Title | 1 | | Identify the report as a systematic review. | 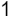 | | |
| **ABSTRACT** | | | |  | | |
| Abstract | 2 | | See the PRISMA 2020 for Abstracts checklist. | 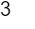 | | |
| **INTRODUCTION** | | | |  | | |
| Rationale | 3 | | Describe the rationale for the review in the context of existing knowledge. | 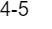 | | |
| Objectives | 4 | | Provide an explicit statement of the objective(s) or question(s) the review addresses. | 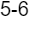 | | |
| **METHODS** | | | |  | | |
| Eligibility criteria | 5 | | Specify the inclusion and exclusion criteria for the review and how studies were grouped for the syntheses. | 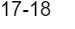 | | |
| Information sources | 6 | | Specify all databases, registers, websites, organisations, reference lists and other sources searched or consulted to identify studies. Specify the date when each source was last searched or consulted. | 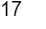 | | |
| Search strategy | 7 | | Present the full search strategies for all databases, registers and websites, including any filters and limits used. | 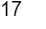 | | |
| Selection process | 8 | | Specify the methods used to decide whether a study met the inclusion criteria of the review, including how many reviewers screened each record and each report retrieved, whether they worked independently, and if applicable, details of automation tools used in the process. | 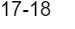 | | |
| Data collection process | 9 | | Specify the methods used to collect data from reports, including how many reviewers collected data from each report, whether they worked independently, any processes for obtaining or confirming data from study investigators, and if applicable, details of automation tools used in the process. | 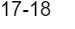 | | |
| Data items | 10a | | List and define all outcomes for which data were sought. Specify whether all results that were compatible with each outcome domain in each study were sought (e.g. for all measures, time points, analyses), and if not, the methods used to decide which results to collect. | 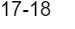 | | |
|  | 10b | | List and define all other variables for which data were sought (e.g. participant and intervention characteristics, funding sources). Describe any assumptions made about any missing or unclear information. | 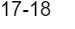 | | |
| Study risk of bias assessment | 11 | | Specify the methods used to assess risk of bias in the included studies, including details of the tool(s) used, how many reviewers assessed each study and whether they worked independently, and if applicable, details of automation tools used in the process. | 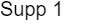 | | |
| Effect measures | 12 | | Specify for each outcome the effect measure(s) (e.g. risk ratio, mean difference) used in the synthesis or presentation of results. |  | | |
| Synthesis methods | 13a | | Describe the processes used to decide which studies were eligible for each synthesis (e.g. tabulating the study intervention characteristics and comparing against the planned groups for each synthesis (item #5)). | 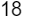 | | |
|  | 13b | | Describe any methods required to prepare the data for presentation or synthesis, such as handling of missing summary statistics, or data conversions. | 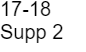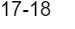 | | |
|  | 13c | | Describe any methods used to tabulate or visually display results of individual studies and syntheses. | 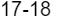 | | |
|  | 13d | | Describe any methods used to synthesize results and provide a rationale for the choice(s). If meta-analysis was performed, describe the model(s), method(s) to identify the presence and extent of statistical heterogeneity, and software package(s) used. | 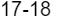 | | |
|  | 13e | | Describe any methods used to explore possible causes of heterogeneity among study results (e.g. subgroup analysis, meta-regression). | 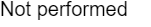 | | |
|  | 13f | | Describe any sensitivity analyses conducted to assess robustness of the synthesized results. | 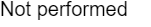 | | |
| Reporting bias assessment | 14 | | Describe any methods used to assess risk of bias due to missing results in a synthesis (arising from reporting biases). |  | | |
| Certainty assessment | 15 | | Describe any methods used to assess certainty (or confidence) in the body of evidence for an outcome. | 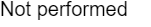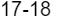 | | |
| **Section and Topic** | | | **Item #** | **Checklist item** | | **Location where item**  **is reported** |
| **RESULTS** | | | | | |  |
| Study selection | | | 16a | Describe the results of the search and selection process, from the number of records identified in the search to the number of studies included in the review, ideally using a flow diagram. | | 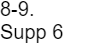 |
|  |  |  | 16b | Cite studies that might appear to meet the inclusion criteria, but which were excluded, and explain why they were excluded. | | 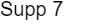 |
| Study characteristics | | | 17 | Cite each included study and present its characteristics. | | 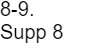 |
| Risk of bias in studies | | | 18 | Present assessments of risk of bias for each included study. | | 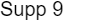 |
| Results of individual studies | | | 19 | For all outcomes, present, for each study: (a) summary statistics for each group (where appropriate) and (b) an effect estimate and its precision (e.g. confidence/credible interval), ideally using structured tables or plots. | | 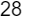 |
| Results of syntheses | | | 20a | For each synthesis, briefly summarise the characteristics and risk of bias among contributing studies. | | 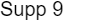 |
|  |  |  | 20b | Present results of all statistical syntheses conducted. If meta-analysis was done, present for each the summary estimate and its precision (e.g. confidence/credible interval) and measures of statistical heterogeneity. If comparing groups, describe the direction of the effect. | | 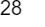 |
|  |  |  | 20c | Present results of all investigations of possible causes of heterogeneity among study results. | |  |
|  |  |  | 20d | Present results of all sensitivity analyses conducted to assess the robustness of the synthesized results. | | 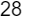 |
| Reporting biases | | | 21 | Present assessments of risk of bias due to missing results (arising from reporting biases) for each synthesis assessed. | | 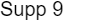 |
| Certainty of evidence | | | 22 | Present assessments of certainty (or confidence) in the body of evidence for each outcome assessed. | | 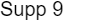 |
| **DISCUSSION** | | | | | |  |
| Discussion | | | 23a | Provide a general interpretation of the results in the context of other evidence. | | 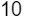 |
|  |  |  | 23b | Discuss any limitations of the evidence included in the review. | | 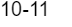 |
|  |  |  | 23c | Discuss any limitations of the review processes used. | | 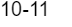 |
|  |  |  | 23d | Discuss implications of the results for practice, policy, and future research. | | 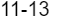 |
| **OTHER INFORMATION** | | | | | |  |
| Registration and protocol | | | 24a | Provide registration information for the review, including register name and registration number, or state that the review was not registered. | | 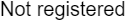 |
|  |  |  | 24b | Indicate where the review protocol can be accessed, or state that a protocol was not prepared. | | 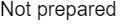 |
|  |  |  | 24c | Describe and explain any amendments to information provided at registration or in the protocol. | | 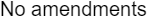 |
| Support | | | 25 | Describe sources of financial or non-financial support for the review, and the role of the funders or sponsors in the review. | | 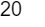 |
| Competing interests | | | 26 | Declare any competing interests of review authors. | | 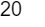 |
| Availability of data, code and other materials | | | 27 | Report which of the following are publicly available and where they can be found: template data collection forms; data extracted from included studies; data used for all analyses; analytic code; any other materials used in the review. | | 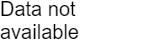 |


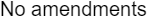

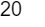

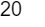
*From:* Page MJ, McKenzie JE, Bossuyt PM, Boutron I, Hoffmann TC, Mulrow CD, et al. The PRISMA 2020 statement: an updated guideline for reporting systematic reviews. BMJ 2021;372:n71. doi: 10.1136/bmj.n71

For more information, visit: <http://www.prisma-statement.org/>
